# [Corrigedum] miR‑185 inhibits non‑small cell lung cancer cell proliferation and invasion through targeting of SOX9 and regulation of Wnt signaling

**DOI:** 10.3892/mmr.2023.12984

**Published:** 2023-03-23

**Authors:** Zhengwen Lei, Hongcan Shi, Wei Li, Duonan Yu, Feiyang Shen, Xi Yu, Dan Lu, Chao Sun, Kai Liao

Mol Med Rep 17: 1742–1752, 2018; DOI: 10.3892/mmr.2017.8050

Subsequently to the publication of the above paper, an interested reader drew to the authors’ attention that, for the Transwell invasion assay experiments with the SK-MES-1 cell line shown in Fig. 4A on p. 1748, the ‘mimic'NC’ and ‘inhibitor-NC’ data panels showed overlapping sections, such that these data may have been derived from the same original source even though they were intending to show the results of different experiments.

The authors have consulted their original data, and realize that the ‘inhibitor-NC’ data panel was inadvertently selected incorrectly for Fig. 4A. The revised version of Fig. 4, showing the correct data for the ‘inhibitor-NC’ experiment, is shown on the next page. Note that the error made during the assembly of Fig. 4 did not significantly affect either the results or the conclusions reported in this paper, and all the authors agree to this Corrigendum. The authors are grateful to the Editor of *Molecular Medicine Reports* for allowing them the opportunity to publish this corrigendum, and apologize to the readership for any inconvenience caused.

## Figures and Tables

**Figure 4.**
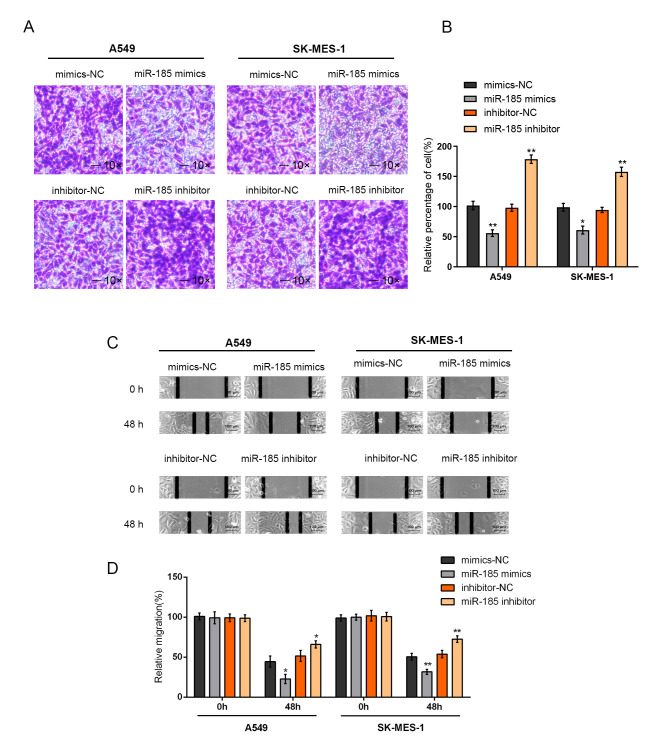
Effects of miR-185 on non-small cell lung cancer cell invasion and migration. A549 and SK-MES-1 cells were transfected with miR-185 mimics or miR-185 inhibitor to achieve ectopic miR-185 expression or miR-185 inhibition. The cell invasion capability of A549 and SK-MES-1 cells was determined using Transwell assays. (A) Representative images and (B) statistical analysis are presented. The cell migration capability of A549 and SK-MES-1 cells was determined using a cell scratch test. (C) Representative images and (D) statistical analysis are presented. The data are presented as the mean ± standard deviation of three independent experiments. *P<0.05, **P<0.01 vs. respective mimics-NC group. NC, negative control; miR, microRNA.

